# Sport supplement use among high school rugby players in South Africa: A scoping review

**DOI:** 10.17159/2078-516X/2022/v34i1a13348

**Published:** 2022-01-01

**Authors:** B Harmse, H Noorbhai

**Affiliations:** Department of Sport and Movement Studies, Faculty of Health Sciences, University of Johannesburg, Johannesburg, South Africa

**Keywords:** doping, ergogenic aids, nutrition, youth, athletes

## Abstract

**Background:**

The use of sport supplements has increased for all types and levels of sport, with an estimated increase of 5.8% annually. Sport supplement usage and doping among high school athletes has increased over the years to meet the demands of the sports.

**Objective:**

This scoping review identifies the trends and gaps in current literature regarding sport supplement use among high school rugby players in South Africa.

**Methods:**

A search was conducted using six electronic databases, namely Oxford Academic, Emerald Publishing, ResearchGate, SABINET, PubMed and Google Scholar. The eligibility of the articles was determined by means of a PRISMA flow diagram with the following inclusion criteria: (1) research concerning supplement use among rugby players, (2) research concerning supplement use among high school rugby players. Five articles all comprising of cross-sectional study designs were included in this scoping review.

**Results:**

The prevalence of sport supplement use among adolescent rugby players ranged from 30% to 45%. Protein supplements (31% – 79%) were the most commonly identified supplement used among adolescent rugby players with the aim to improve sport performance as the most common reason for use. The internet (74%) and magazines (72%), followed by coaches comments (28% to 30%), were given as the most common sources of information.

**Conclusion:**

The use of sport supplements is increasing among high school athletes due to the belief that these substances will provide sporting performance benefits or enhance the competitive ‘edge’ of these athletes. Additional education regarding the safety of supplements is necessary to lower the incidences of doping among young athletes and avoid the adverse health effects that uncontrolled supplement use can cause.

Rugby is a popular sport worldwide with an estimated 8.5 million players globally. The popularity of this sport is ever-increasing. ^[1]^ The popularity of this sport has extended to the South African youth with an estimate of 388 393 registered adolescent players under the age of 18 years old. ^[2]^ Rugby is a contact team sport comprised of 15 players in various positional roles, namely: full-back, wing, centre, fly-half, scrum-half, number 8, flankers, hooker, props and locks. ^[3]^

The professionalisation of the sport that occurred during the 1990s has led to the significant increase in the level of competition between players, and this mindset has extended to the sporting level of the youth resulting is highly competitive high school rugby development. ^[1,4]^ This professionalisation has resulted in many young athletes aspiring to achieve certain body characteristics, such as increasing muscle size and strength to keep up with the demands of the sport. ^[4]^ This has related to the saying ‘bigger is better’, as a rugby player with significant body mass is believed to have greater influence on the game in terms of strength, speed and power. ^[2]^

According to the International Olympic Committee (IOC), a sport supplement is defined as any substance that is consumed by athletes for the purpose of improving their sporting performance. ^[5]^ Many young athletes consume sport supplements for a variety of reasons, some of which include the desire to increase their muscle mass and body size or gain benefits in sport performance or strength. ^[6]^ It is of primary concern that an athlete is aware of the risks associated with the use of sport supplements, such as health risks or legal ramifications linked to doping. ^[7]^

The term ‘doping’ can be defined as the use of substances by athletes to gain a performance benefit. However, most of these substances are classified as prohibited substances by the World Anti-doping Agency (WADA). ^[8]^ Doping by athletes can be done purposefully; however, there are some athletes who unknowingly consume prohibited substances and this is regarded as un-intentional doping. ^[5]^ For a substance to be seen as a prohibited substance according to WADA, it needs to be a substance that has been classified with performance-enhancing benefits, risks the athlete’s health and does not maintain the code of WADA regarding the spirit of sport. ^[9]^

The sport supplement industry has been criticised due to its poorly regulated production of sport supplements which possibly lead athletes to unintentionally doping, and subsequently failing to comply with WADA. A review of the available literature regarding the general prevalence of sport supplements used by adolescent rugby players and the general attitudes towards sport supplement use in South Africa is imperative for investigation.

## Review of the current literature

### Prevalence of sport supplement use in athletes

Globally, the use of sport supplements has increased by 5.8% annually in all types and levels of sport. ^[7]^ Approximately 30% to 95% of athletes consume a sport supplement. The broad percentage range is due to the variations in the different types of athletes in terms of the sport, the level of play and demographical information. However, this particular study identified that 65% of the participants who play rugby for the Spanish Rugby Federation consumed sport supplements. ^[10]^ Studies performed in South Africa concerning the use of sport supplements have shown that an estimated 40% to 70% of the athletes indicated that they consume sport supplements. ^[11]^ A study conducted in Germany found that the majority (91%) of elite German athletes consumed sport supplements, while in a study conducted on Kenyan Rugby League players, 15% of the players indicated that they consume dietary supplements. ^[12, 13]^

### Common types of sport supplements used by athletes

Research shows that there is a variety of different supplements that athletes use depending on their sport and level of training. ^[5]^ When comparing the predominant sport supplement consumed by professional rugby players in Europe, it was found that the most common sporting supplement used by rugby players from the European Super League, were protein supplements followed by creatine (64%), 56% of the participants used carbohydrate supplements, and only 36% used caffeine as a type of sport supplement. ^[14]^ Whey protein and caffeine supplements were the predominant sport supplements consumed by rugby players in the Spanish Rugby Federation, namely 44% and 42%, respectively. This is followed by energy supplements (energy drinks and bars) which ranged from 34% to 38% of the participants. ^[10]^ When considering rugby players on the African continent, a study of rugby player participation taking part in the Kenyan Rugby League, 50% of the players used creatine monohydrate as a sport supplement followed by a whey protein supplement (33%). ^[13]^ In a study on the common types of sport supplements consumed by the under-21 Blue Bulls rugby players in South Africa, it was found that the most common sport supplement consumed them was protein supplements (50%) followed by amino acids (31%). ^[15]^

### Common sources of information regarding the use of sport supplements in athletes

According to a study by McCreanor et al. the most common source of information for sport supplements is the internet. ^[16]^ A sport trainer was the most popular source of information regarding the use of sport supplements in a study conducted by Sánchez-Oliver et al. ^[10]^ The use of scientific literature as a source of information is low. This is problematic as it indicates that athletes are not referring to scientifically based information regarding the use of supplements. ^[17]^

### Common reasons for supplement use in athletes

In a study conducted by Sánchez-Oliver et al. ^[10]^, the main reason for sport supplement usage in rugby players was to enhance their sporting performance during training and during matches, stated by 62% of the participants. It is possible that many young athletes make use of sport supplements due to poor body image or body satisfaction, which can be linked to the desire to use these sport supplements to increase body size and muscle strength to meet the demands of the sport that they participate in. ^[6]^ When questioned why the athletes used the sport supplements, 42% of the rugby players who were part of the Kenyan Rugby League felt that the sport supplements aided their performance. However, 45% of the surveyed participants indicated that the sport supplements did not influence their sporting performance, while 43% of the players indicated that they felt using sport supplements was unnecessary. ^[13]^ All the under-21 Blue Bulls rugby players who used supplements in the study, indicated that the main reason for them using sport supplements was to increase the size of their muscles, followed by increasing their energy levels post-training or post-match (38%). ^[15]^

### General attitudes concerning sport supplements usage in athletes

In a study conducted on rugby players who participated in the European Super League, it was found that the majority of the players were aware of the associated risks when using sport supplements. This information was contradicted when asked if they believed that the sport supplements that they regularly used were safe and correctly tested. ^[14]^ A study in France indicated that 90% of elite athletes viewed doping as an act of dishonesty and that it is dangerous for one’s health. ^[9]^

Education regarding supplement use is a vital part of an athlete’s training with an increase in the desire to use sport supplements. However, there is limited emphasis on the need to improve education for young athletes to make informed decisions about sport supplements for the sake of their health and sporting careers. ^[18]^

In summary, education concerning sport supplements is vital not only to protect athletes against the risks from supplements but to also allow the athletes make informed decisions concerning the use of these supplements. Educating athletes regarding optimal nutritional knowledge and maturation can lead to sport success instead of them risking the use of prohibited substances.

## Methods

This scoping review made use of the Preferred Reporting Items for Systemic Reviews and Meta-Analyses (PRISMA) guidelines method to identify relevant articles. ^[19]^ A search was conducted to identify possible relevant studies using six databases (Oxford Academic, Emerald Publishing, ResearchGate, SABINET, PubMed and Google Scholar), as well as two additional resources (South African Journal of Sports Medicine and the British Journal of Sports Medicine). The search was conducted on the 16^th^ September 2021. During the search for relevant articles, keywords such as ‘sport supplements’, ‘doping’, ‘adolescent athletes’, ‘high school rugby players’, as well as phrases such as ‘doping in athletes’, ‘doping in adolescents’, ‘doping in adolescent athletes’, ‘sport supplement use in athletes’, ‘sport supplement use in adolescent athletes’, ‘sport supplement use in rugby players’, and ‘doping in rugby players’ were applied. In addition, the search was limited to studies that included rugby players who used sport supplements.

### The inclusion criteria for this review

- Studies that included research concerning supplement use among rugby players.
- Studies that included research with sources of information concerning supplement use among rugby players.
- Studies that included research concerning supplement use among adolescent/high school rugby players.

### The exclusion criteria for the articles obtained for this review

- Articles removed based on unrelated topics/titles (articles that did not include supplement use in adolescent athletes).
- Articles that had a meta-analysis methodology.
- Articles that were published in a language other than English.

**Fig. 1:**
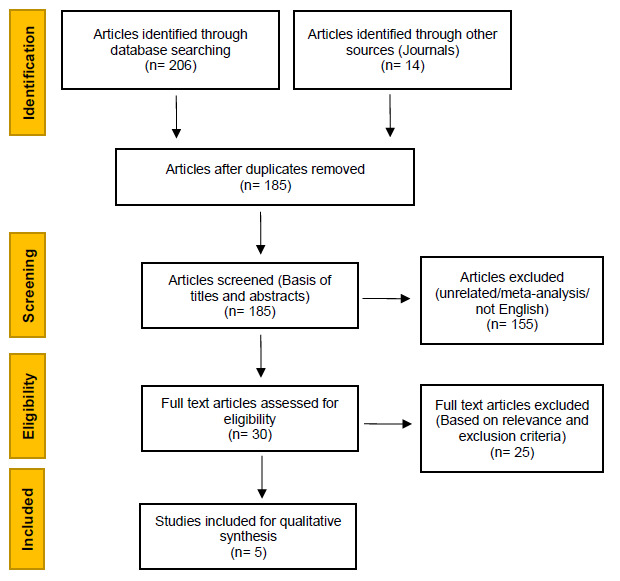
Schematic of the process followed for the literature search according to the PRISMA guidelines

Once articles were identified from the above-mentioned databases, all duplicates retrieved were removed. All articles were screened and those not meeting the inclusion criteria were removed. The screening process was completed by means of the PRISMA method guidelines shown in Figure 1. ^[19]^ In order to extract the relevant data from the eligible sources, the authors’ initials and surnames, year of publication, population size, age of the participants, prevalence of sport supplement use, common reasons for the use, common types of supplement used among rugby players, sources of information and the general attitudes and knowledge of the athletes regarding supplement usage among high school rugby players in South Africa were identified. These were performed for each of the selected studies and tabulated individually according to the topic. The studies used in this scoping review were all conducted in South Africa.

### Data synthesis

If the data present in the article met the inclusion criteria requirements, data related to the prevalence, use and attitudes towards sport supplement use among high school athletes was extracted by the author and set up in a Microsoft Excel chart (2016). Information on the authors, date of publication, population size, prevalence of sport supplement use, common sport supplements used, common reasons for sport supplement use and key findings regarding the general attitudes towards supplement use and doping were extracted by the author and recorded. The recorded data was tabulated according to different topics and presented in the Results section.

## Results

A total of 206 possible relevant articles were identified by searching six databases and 14 possible relevant articles were identified from two additional journals. The articles were tabulated and duplicate articles were removed. After the duplicate articles were excluded, 185 articles were screened by the titles and abstract to determine relevance of the selected articles of which 155 articles were excluded due to not meeting the inclusion criteria. A total of 30 full-text articles were screened for eligibility and the resulting articles used in this review were narrowed down to five articles.

### Study design

All the articles selected for this review were of a descriptive, quantitative, cross-sectional study design by means of self-administered surveys distributed to the high school rugby players. One study was conducted for a period of four years (2009 to 2012), where participants were approached once a year at an annual rugby training camp.

### Demographic information and location of studies

The age of the participants in the selected articles was between 15 to 18 years. All the participants played rugby at high school level, and were generally in the first or second teams of their high schools. Half of the studies (n = 2) took place in the Gauteng province of South Africa, a single study took place in the Western Cape province of South Africa, another study took place in KwaZulu Natal, with the remaining study including participants across different regions in South Africa.

### Prevalence of sport supplement usage

According to the identified studies (Table 1), the prevalence of supplement use among the participants ranged between 30% and 45%, with one study indicating that 3.9% of the participants used banned/prohibited sport supplements. One study did not identify a specific number of rugby players who made use of sport supplements as a separate topic. However, the prevalence of the different types of supplements used by the athletes were described in the study.

### Type of supplements used by athletes and reasons for their usage

There were varying types of commonly used supplements used by athletes according to the type of supplements listed in the surveys of the studies. Table 1 summarises the most common supplements used by rugby players in the reviewed studies. In the first study by Gradidge et al. ^[20]^, the most common supplement used (classified as a banned substance) was a growth hormone, where 5% of the participants indicated that they made use of this supplement. The second most commonly prohibited substances used by high school athletes was adrenaline and anabolic steroids, and 4% of participants used these banned substances, respectively. When surveyed regarding the use of unbanned sport supplements, 61% of the participants indicated that they used protein supplements as well as vitamin supplements. Creatine supplement use was the least popular supplement used among high school athletes in Johannesburg. The study conducted by Nolte et al. ^[21]^ identified that 29% of the participants indicated that they used dietary supplements, however, no specific supplements were indicated. Almost a quarter of the participants (24%) indicated that they use anabolic steroids. The use of protein supplements was the most commonly reported supplement used among high school athletes, with 31% of the participants stating that they use these supplements, based on the review study by Duvenage et al. ^[22]^ In the study conducted by Jooste ^[23]^, carbohydrate supplementation was the most commonly used sport supplement among rugby players (92%), followed by the use of protein supplements (79%). Creatine (37%) and glutamine (37%) were the least common supplements used by the rugby players. High school rugby players in KwaZulu-Natal indicated that the most common supplement used among the team players were protein supplements (43%) while the least common sport supplement consumed was strength boosting supplements and the supplement nitric oxide (3%). ^[24]^

Only three studies included questions regarding the most common reasons for rugby players making use of the sport supplements. The majority of the participants indicated that they used them to improve or enhance their sporting performance with a total of 68% of the participants in a study conducted by Gradidge et al. ^[20]^. The least common reason for using the supplement was the fear of not considered being part of the rugby team. When questioned on the reason for using specific supplements, the most common reason for the use of carbohydrate supplements was to lower the risk of fatigue and shorten the recovery time after play. The most common reason for using protein supplements among high school rugby players was to increase muscle size and muscle strength in order to improve sporting performance. ^[23]^ In the study conducted on high school rugby players in KwaZulu-Natal, the most common reasons for using protein and creatine supplements was to increase the strength of the muscles and the muscle mass of the athletes, resulting in athletes increasing in body size. ^[24]^

### Source of information regarding sport supplements emanating from research studies

The least common source of information regarding sport supplements that adolescent rugby players consulted regarding their use and safety were dieticians, with one study indicating that 4% of the participants consulted a dietician (Table 1). In another study, the participants indicated that the internet (74%) and magazines (72%) were the sources that they relied on to obtain information regarding using sport supplements. In two studies, coaches were the most common source of information for supplement use, with up to 30% of the participants in the study by Nolte et al. ^[21]^ and 28% of the participants in the study by Jooste ^[23]^ indicating that they approached their coach for information. Only 16% of the participants in the study conducted by Gradidge et al. ^[20]^ indicated that they approached a biokineticist for information about the use of sport supplements. The most common source of information among rugby players in KwaZulu-Natal was information from the player’s friends. ^[24]^

### General attitudes towards sport supplements

In the study conducted by Gradidge et al. ^[20]^, 91% of the participants indicated that they felt the use of sport supplements among high school athletes is increasing in South Africa with 37% of the rugby players surveyed believing that their use is on the rise while the majority (84%) felt that learners are being pressured into using these supplements in high school sport. Thirty-seven percent of the participants agreed that they felt tempted to use performance enhancing supplements; however, 27% of them said that they never considered using sport supplements noted during the review of the study by Gradidge et al. ^[20]^ A similar view was stated in the study by Nolte et al. ^[21]^ with 39% of the participants indicating that they would consider using supplements (non-prohibited) to improve sporting performance, and more than half (70%) indicated they would not use banned/prohibited substances to improve sporting performance. When considering specific types of sport supplements used by athletes, 68% of the participants believed that they would use a protein supplement for the purpose of improving muscle size. ^[22]^ In a study by Jooste ^[23]^, players stated that they were more likely to use carbohydrate supplements (91%), followed by protein supplements (90%), glutamine (59%) and creatine (57%) in the future.

Based on the study by Gradidge et al. ^[20]^, 61% of the participants indicated that they felt that the use of sport supplements was unethical, which is similar to the view by the participants in the study by Nolte et al. ^[21]^ The majority of the participants (85%) stated that prohibited substances were harmful to one’s health while 84% indicated that using banned substances to improve performance was morally wrong. However, it was noted in the study by Gradidge et al. ^[20]^ that the majority of the participants (91%) indicated that they believed that the selling of banned substances should not be banned in sport. More than half (59%) of the participants in the study by Nolte et al. ^[21]^ indicated that there is not sufficient education on the safety and dangers regarding the use of sport supplements, particularly banned substances. This finding was, however, contradicted in the study by Gradidge et al. ^[20]^ where 81% of the participants indicated that there is no requirement for more education on the use of prohibited substances and doping. In the same study, 91% of the participants stated that there is no requirement for stricter ramifications or punishments for doping in sport.

In the study conducted by Gradidge et al. ^[20]^, almost three-quarters (72%) of the participants stated that they did know which substances were banned according to WADA, while according to the study by Nolte et al. ^[21]^, 74.8% of the participants felt that they were well-informed about the substances classified as banned in their sport. More than half of the participants (55%) indicated that the use of regular doping control testing in high school sport would lower the instance of prohibited supplement use, and only 48% of the participants indicated that they believed more tests for doping should be conducted. In the study conducted by Duvenage et al. ^[22]^, 60% of the participants stated that they believed that using sport supplements will not lead to a positive doping control test.

Overall, knowledge regarding the safety and correct labelling of supplements and the belief that supplements are the best way to achieve bigger muscles was considered poor (43%). ^[23]^ Only 48% of the players agreed that natural sources of protein found in food is better than that found in supplements; however, there is a similar average in the study by Duvenage et al. ^[22]^ with 40% of the participants believing that the source of protein found in foods is of a higher quality than that found in sport supplements. Thirteen percent of the athletes agreed that muscle mass gain is not linked to creatine supplementation. Less than 5% of the players agreed that glutamine supplements are not effective in sport performance for athletes. ^[24]^ Twenty-two percent of the high school rugby players indicated that they would try a banned substance to improve their performance. ^[23]^

## Discussion

This scoping review identified that there are very few studies conducted on sport supplement use among high school rugby players in South Africa. The use of sport supplements with the aim to improve performance is an age-old tale that can be traced back to the third century BC, where evidence was found that the athletes of that era made use of substances that improved their sporting performance, with additional information identifying that supplement use was prevalent among Greek Olympians in 776 BC. ^[7, 25]^

As mentioned, sport supplements are often used by athletes to improve sport performance, and different sport supplements have different physiological effects and adaptations that result in improvements in sport. When considering the most prevalent sport supplements used by athletes in this scoping review, protein supplements were used to improve sport performance by increasing muscle size and strength. Protein supplements provide increases in protein synthesis and lowers the rate of degeneration of proteins in the skeletal muscles. ^[26, 27]^ The increase in protein synthesis results in increases in muscle size and strength which would be beneficial to athletes, particularly rugby players, to meet the requirements of the sport. ^[26]^ Carbohydrate supplements are generally used by athletes to increase their energy before, during and after training, as well as for pre- and post- match energy. In addition, caffeine is a stimulating substance that increases central nervous systems activation. However, caffeine is linked to increases in a person’s heart rate and blood pressure, and may result in an increased sense of anxiety and sleep abnormalities which may negatively impact athletes’ sporting performance. ^[27, 28]^ The use of banned substances such as anabolic steroids amongst high school athletes was identified in the study by Nolte et al. ^[21]^ where 24% of the participants used this banned substance and 4% in the study by Gradidge et al. ^[20]^ Anabolic steroids have similar physiological effects on the body as protein supplements; however, anabolic steroids are a form of synthetic testosterone that increase protein synthesis, and as a consequence, increases lean body mass and muscle strength. Anabolic steroids have many side effects that athletes are not always aware of, such as increases in low-density lipoproteins and mood disturbances, typically aggression. ^[27]^ Sport supplements may have some benefits to sport performance; however, athletes should be aware of the side effects and how these supplements influence the body before deciding to use them.

### Prevalence of sport supplements

Supplementation with the aim to enhance sporting performance has continued to increase over the decades in all sports with an estimated range of between 30% to 95% of athletes making use of a form of supplementation globally. ^[10]^ This wide range of the prevalence of sport supplements is dependent on the type of sport, the level of competition and the physical requirements needed to succeed in the sport in terms of strength, speed and agility, which is particularly prominent in sports such as rugby. ^[26]^ This demand and the professional level of the sport often leads to young athletes seeking additional sources to improve their sporting performance and generally sport supplements are the first line of action to achieve this. ^[25]^

It appears as if high school athletes, together with parents and coaches, have had a change in mind-set from playing for the love and enjoyment of the sport to athletes striving to excel in their sport in order to be the best player. This often leads the athletes to use sport supplements without always considering the safety and regulation of these supplements. ^[20, 24, 25]^ This is supported by evidence identified in the studies reviewed where the estimate range of sport supplement usage among high school athletes in South Africa range from 30% to 54%.

### Common types of sport supplements and reasons for their use

The increased use of sport supplements has funnelled down to high school level sports in South Africa (particularly in rugby) where there’s a desire among athletes to increase strength or change their body size to be a better player depending on the their position in their sport. ^[15]^ Although some sport supplements may be ergolytic, they can also be ergogenic in which case they enhance exercise capacity and/or athletic performance. The continual rise of sport supplements usage may be attributed to some degree to body image dissatisfaction that is present in young adolescents as well as young athletes. This trend was evident in the study conducted by Yager and O’Dea ^[6]^ where only 30% of the athletes were satisfied with their body image and the remaining athletes indicated that they would want to be either larger or smaller. In the review of the study conducted by Gradidge et al. ^[20]^, the majority of athletes believed that the use of sport supplements in high school athletes is increasing in South Africa. This trend is evident when considering the reasoning behind the use of sport supplements among high school rugby players, particularly in South Africa, where 68% of the participants who took part in the survey conducted by Gradidge et al. ^[20]^ indicated that they used supplements to improve their sporting performance. In the review of the study by Strachan ^[24]^, it was noted that more than half the rugby players (54%) decided to use protein supplements to increase their strength in order to play their sport.

### Common sources of information regarding sport supplements

Advertising of sport supplements (whether on television, the internet or in magazines) may also contribute to the increase in their use due to the ‘hype’ created by using well-known athletes (or social media producers) by supposedly providing benefits of such supplements. However, these advertisements often include information that is biased in order to convince athletes to use the supplements and these athletes or coaches use this information to confirm the safety and benefits of the supplements. ^[24]^ Athletes who use this information (which is often not scientifically sound) or approach coaches or teammates who have little knowledge of supplements may lead to the misuse of the supplements by using more of the recommended amount, which in turn can lead to health problems. This misuse is worsened by athletes not having sufficient knowledge to make informed decisions when reading the ingredients lists. ^[5,29]^

Improving the general sources of information regarding sport supplements is essential as poor sources of information may lead to poor decisions regarding their use. It was identified that sources such as the internet, along with information from coaches and friends, were seen as the most common information sources used by high school athletes (in the studies reviewed). A range of 28% to 60% of athletes indicated that they consulted their sporting coach regarding the information concerning these sport supplements. This evidence is supported by a study conducted on high school rugby players in Ireland where 66.9% indicated that they consulted with their coach regarding this matter. ^[30]^ The internet may be considered a beneficial source of information in terms accessibility and amount of information available. However, most sources on websites are not scientifically reviewed or correct which may lead to misinformation. ^[25]^

The use of scientific literature and knowledge of qualified healthcare practitioners, such as dieticians, are often not the first line of consultation when considering the use of sport supplements, with only 4% of rugby players in the Western Cape ^[23]^ indicating that they approached a qualified dietician for information. A similar finding is also found in the study by Walsh et al. ^[30]^ where only 10 of the 203 rugby players consulted a qualified healthcare professional. This can be regarded as problematic in terms of obtaining the correct information on the safe use of sport supplements, as sources often used by athletes may be unreliable or not evidence-based, which may result in athletes using supplements that may be detrimental to their health and well-being.

### General attitudes towards the use of sport supplements

There are often discrepancies between the general attitudes regarding the use of sport supplements and doping which may be linked to the educational gap as many players reported that more education of dietary requirements and supplementation are necessary.

In the study conducted by Walsh et al. ^[30]^, the majority of the adolescent rugby players felt that additional education regarding nutrition and the use of sport supplements would be beneficial as the participants in this study showed a poor understanding and knowledge about the correct nutrition for their age and the demands of their sport. A similar view was expressed by the athletes surveyed in the study by Nolte et al. ^[21]^ where 59% of the participants felt that more education was required. However, this view was not shared by athletes in Johannesburg high schools where majority of the athletes (81%) felt that there was enough education available regarding the use of these sport supplements. ^[20]^

The risk of doping is further increased due to the gap present in the knowledge of labelling and the identification of harmful ingredients which may lead to unintentional doping through the use of banned substances. The risk is also considered higher if the athlete’s attitude towards doping does not deter him/her not to make use of these supplements to improve performance or their body image. In the review of the study by Duvenage et al. ^[22]^, more than half the participants (60%) believed that the use of banned substances or doping substances would not result in the athlete testing positive for doping. It was found that majority of the athletes that were part of the studies reviewed had a negative attitude towards the idea of the using banned substances. Some athletes indicated that it was against good morals and was, therefore, considered unethical.

Protocols in dealing with punishments ranging from the removal of medals or personal/world records to a more severe punishment of suspending athletes from partaking in the sport for a period of time have been implemented by WADA. ^[5]^ This level of punishment has led to many athletes believing that this is an effective means of deterring athletes from doping. Athletes who were surveyed in the study by Gradidge et al. ^[20]^ indicated that there was no need to increase the severity of the punishment linked to doping. According to the athletes surveyed in the study by Duvenage et al. ^[22]^ less than half of the participants believed that more testing should be conducted in schools. However, 14% of the under-16 rugby players in South Africa indicated that if there was a chance that they would not be caught by a positive doping control test, and they would consider using a banned substance. ^[22]^ This shows that athletes at a high school level are somewhat fearful of the consequences of being caught for doping but would still consider doping if there was a chance of not getting caught.

The increase in usage of sport supplements in high school athletes is a matter of concern as the sport supplements industry is often under scrutiny due to the poor regulation of the production and the labelling of the products. ^[7]^ This is evident is the article by the International Olympic Committee where it was found that athletes who have been tested for doping and had a positive test, made use of sporting supplements that may have contained small amounts of prohibited substances which were not indicated on the product’s list of ingredients resulting in unintentional doping. ^[21]^ This may support the ‘gateway hypothesis’ where athletes using sport supplements may use other substances that may be banned, such as anabolic steroids, in the future thereby opening the door for positive doping control tests and dangers to the athlete’s health. ^[23]^ When surveyed regarding the knowledge of banned substances and the likely adverse side effects to the athlete and therefore meets the criteria according to WADA for being a banned substance. ^[9]^ Seventy-two percent of the athletes in South Africa in the study reviewed by Gradidge et al. ^[20]^ indicated that they were aware of what were banned substances according to WADA, with a similar view in the study conducted by Nolte et al. ^[21]^. This would be considered as a positive attitude towards anti-doping awareness. However, it is important to note that in the same study^[21]^, 3.9% of the surveyed athletes indicated that they currently use a form of banned substance, with 24% of the athletes stating that they had used anabolic steroids as a form of supplementation.

The gap in education concerning sport nutrition and performance needs to be addressed in order for athletes to meet the recommended dietary requirements to reduce the need of sport supplements. ^[30]^ It appears that many athletes make use of sport supplements as a means of substituting the missing nutrients of their diet. Globally, many athletes believe that obtaining the necessary nutrients to perform well at sport can only be achieved through supplementation. This is a perception often supported by the parents of the athletes (as indicated by the parents of athletes in the East Rand of Johannesburg, South Africa). ^[11, 30]^ This evidence is present in the viewpoints of the studies reviewed in this scoping review where less than half of the athletes surveyed stated that natural sources of nutrients (particularly protein sources) are deemed superior to that found in supplements. ^[22]^

### Limitations of the study

In the present scoping review, there were very limited sources reviewed concerning the prevalence and general attitudes regarding the use of sport supplements among high school rugby players in South Africa. However, findings from this review provides some insight into the general attitudes and prevalence of supplement use particularly in Gauteng, Western Cape and KwaZulu-Natal provinces of South Africa.

## Conclusion

The prevalence of sport supplement use is on the rise among all athletes, particularly among high school rugby players. The main reason for the use of sport supplements found in this review was to improve strength and sporting performance. This increase is linked to the heightened demands placed on the athletes by coaches, parents and the athletes themselves to have the ideal competitive ‘edge’ in order to be the best player or to be selected for the best team. An additional factor influencing young athletes to use sporting supplements is the desire to attain a certain physique, such as improved muscles size overcoming the low self-body image that is common among adolescents.

A gap is present in the level of education regarding the use of sport supplements among high school athletes, with athletes believing that sport supplements are a necessary requirement to meet the nutritional needs of their sport. However, little knowledge is available regarding the health risks associated with the use of sport supplements as well as the risks of doping. The internet and coaches are the most commonly used sources of information for athletes regarding supplement use. Information from scientifically reviewed literature and qualified healthcare professionals (such as dieticians) are less frequently consulted. This will lead athletes to believing information that is not reliable regarding the safety of the supplements they use which poses a great health risk with the potential of testing positive for banned substances and risking their sporting career.

However, some athletes would consider using a banned substance if there was no testing and severe punishments associated with doping. Further education should be deemed necessary regarding sport nutrition and the safety of using sport supplements. Improvements in an athlete’s diet will ensure that these athletes achieve the required nutrients to aid their sporting performance. This will assist in decreasing the prevalence of sport supplement use among high school rugby players in particular and lower the overall incidence of doping in athletes.

Further research is required to document the prevalence of sport supplement use on a larger scale, in other countries, and using more studies. Furthermore, a replication of this type of study would also be required in other types of sports in South Africa.

## Figures and Tables

**Table 1:** Sport supplement use, type and sources of information among high school rugby players in South Africa

Author (Year)	Number of Participants	Prevalence of Sport Supplement Use	Common Types of Supplements Used	Source of Information
**Gradidge et al. [20]**	100 (37% rugby players)	30% of the athletes use sport supplements.	Common substances use (prohibited)- growth hormone (5%), anabolic steroids (4%), adrenaline (4%), insulin (2%). Common substances used (non-prohibited)- protein supplements (61%), vitamin supplements (61%), caffeine supplements (57%), carbohydrate supplements (54%), creatine supplements (32%).	Internet (74%), magazines (72%), friends (66%), coach (60%), parent (33%), personal trainer (33%), information brochures (31%), newspapers (29%), pharmacist (24%), TV and school (23%), sibling (19%), biokineticist (16%), books (16%), physician (10%), journals (9%).
**Nolte et al. [21]**	346 (6% rugby players)	45% of the participants indicated that they use sport supplements, 3.9% of the participants indicated that they were using banned substances.	29% of the participants indicated that they use allowed dietary supplements, however no specific supplements were indicated. 24% indicated that they use anabolic steroids.	Coaches (30%), parents (19%), friends (17%), other athletes (16%), South African Institute for Drug-free sport (11%), other (8%).
**Duvenage et al. [22]**	198 (all played rugby)	42% of the participants indicated that they used sport supplements.	Protein (31%), meal replacements/recovery formulas (16%), creatine (16%), carbohydrate (12%), vitamin (10%), pre-workout (5%), glutamine (3%), omega 3/omega 6 fatty acids (3%), non-specific (2%), other (2%), amino acids (1%), fat burners (>1%).	No information regarding the common sources of information used by athletes was present in this study.
**Jooste [23]**	189 (all played rugby)	This study divided the prevalence of supplement use according to the most common type and the difference between two rugby league players.	Carbohydrate (92%), protein (79%), creatine (37%), glutamine (37%).	Coaches (28%), trainer (19%), supplement rep (16%), friends (15%), pharmacist (7%), parents (6%), doctor (4%), dietician (4%).
**Strachan [24]**	68 (all played rugby)	54% of the participants use sport supplements.	Protein (43%), creatine (22%)	Friends (32%), Supplement representatives (22%), Gym (19%), Magazines (19%), Coaches (5%).

## References

[1] Robinson B Pote L Christie C Strength and conditioning practices of high school rugby coaches: A South African context S Afr J Sci 2019 115 9–10 92 97 10.17159/sajs.2019/5837 27075640

[2] Lambert MI Durandt J Long-term player development in rugby–how are we doing in South Africa? S Afr J Sports Med 2010 22 3 67 68 10.17159/2078-516X/2010/v22i3a312

[3] Rugby Football History. Player positions 2007 http://www.rugbyfootballhistory.com/positions.html (accessed 27 October 2021)

[4] Till K Jones B McKenna J The search for size: a doping risk factor in adolescent rugby? Br J Sports Med 2016 50 4 203 204 10.1136/bjsports-2015-094737 25990759

[5] Maughan RJ Burke LM Dvorak J IOC consensus statement: dietary supplements and the high-performance athlete Br J Sports Med 2018 52 7 439 455 10.1136/bjsports-2018-099027 29540367 PMC5867441

[6] Yager Z O’Dea JA Relationships between body image, nutritional supplement use, and attitudes towards doping in sport among adolescent boys: implications for prevention programs J Int Soc Sports Nutr 2014 11 1 13 10.1186/1550-2783-11-13 24670105 PMC3986904

[7] Naidoo K Naidoo R Bangalee V Regulating the South African sport supplement industry: ‘Whey’ overdue S Afr Med J 2018 108 3 166 167 10.7196/SAMJ.2018.v108i3.12961 30004356 PMC6050020

[8] Vlad RA Hancu G Popescu GC Doping in sport, a never-ending story? Adv Pharm Bull 2018 8 4 529 534 10.15171/apb.2018.062 30607326 PMC6311632

[9] Morente-Sánchez J Zabala M Doping in sport: a review of elite athletes’ attitudes, beliefs, and knowledge Sports Med 2013 43 6 395 411 10.1007/s40279-013-0037-x 23532595

[10] Sánchez-Oliver AJ Domínguez R López-Tapia P A survey on dietary supplement consumption in amateur and professional rugby players Foods 2020 10 1 7 10.3390/foods10010007 33375061 PMC7822035

[11] Van der Walt V Coopoo Y A survey of the attitudes and knowledge of parents of high school children on the East Rand on the usage of nutritional supplements S Afr J Sports Med 2016 28 3 74 78 10.17159/2078-516X/2016/v28i3a1674

[12] Diehl K Thiel A Zipfel S Elite adolescent athletes’ use of dietary supplements: characteristics, opinions, and sources of supply and information Int J Sports Nutr Exerc Metab 2012 22 3 165 174 10.1123/ijsnem.22.3.165 22693237

[13] Mse E Kimiywe J Simiyu NWW The extent of dietary supplements use by male rugby players in Kenya J App Biosci 2009 22 1306 1311 [http://hdl.handle.net/10950/488]

[14] Woolfenden A Supplement use in professional rugby league Masters of Philosophy dissertation United Kingdom Liverpool John Moores University 2017 243

[15] Smith VC Dietary intake and supplement use of under 21 rugby players, Blue Bulls South Africa University of the Free State 2007 http:hpplhandle.net/11660/9040

[16] Mc Creanor X Coopoo Y Gabriels G Attitudes towards nutritional supplement gymnasium users in Johannesburg North S Afr J Sports Med 2017 29 1 1 5 10.17159/2078-516X/2017/v29i1a4258

[17] Couture S Lamarche B Morissette E Evaluation of sport nutrition knowledge and recommendations among high school coaches Int J Sports Nutr Exerc Metab 2015 25 4 326 334 10.1123/ijsnem.2014-0195 25386951

[18] van der Bijl P Dietary supplements containing prohibited substances (Part 1) S Afr J Sports Med 2014 26 2 59 61 10.7196/SAJSM.553 24741950

[19] Page MJ McKenzie JE Bossuyt PM The PRISMA 2020 statement: an updated guideline for reporting systematic reviews BMJ 2021 372 10.1136/bmj.n71 PMC8005924 33782057

[20] Gradidge P Coopoo Y Constantinou D Attitudes and perceptions towards performance enhancing substance use in Johannesburg boys high school sport S Afr J Sports Med 2010 22 2 32 36 10.17159/2078-1516X2010/v22i2a313

[21] Nolte K Steyn BJ Kruger P Doping in sport: Attitudes, beliefs and knowledge of competitive high-school athletes in Gauteng Province S Afr J Sports Med 2014 26 3 81 86 10.7196/sajsm.542

[22] Duvenage KM Meltzer ST Chantler SA Initial investigation of nutrition and supplement use, knowledge and attitudes of under-16 rugby players in South Africa S Afr J Sports Med 2015 27 3 67 71 10.7196/SAJSM.8092

[23] Jooste M The prevalence, knowledge and reasons for carbohydrate, protein, creatine and glutamine use among first team rugby players in premier rugby schools in the Western Cape Province [Master of Nutrition] University of Stellenbosch 2016 143

[24] Strachan K Current perceptions and usage practices of nutritional supplements Masters degree (Human Nutrition) University of Stellenbosch 2009 78 https://scholar.sun.ac.za/handle/10019.1/2193

[25] Constantinou D Gradidge P Coopoo Y Prevalence of performance-enhancing substance use by Johannesburg male adolescents involved in competitive high school sports Arch Exerc Health Dis 2011 2 2 114 119 10.5628/aehd.v2i2.102

[26] Kerksick CM Rasmussen CJ Lancaster SL The effects of protein and amino acid supplementation on performance and training adaptations during ten weeks of resistance training J Strength Cond Res 2006 20 3 643 653 10.1519/R-17695.1 16937979

[27] Jenkinson DM Harbert AJ Supplements and sports Am Fam Physician 2008 78 9 1039 1046 19007050

[28] Martin SJ Sherley M McLeod M Adverse effects of sports supplements in men Aust Presc 2018 41 1 10 13 10.18773/austprescr.2018.003 29507454 PMC5828928

[29] Noorbhai MH Gabriels G The enterprising business of nutritional supplements: an eye-opener Int J Drug Dev & Res 2016 8 1 42 43

[30] Walsh M Cartwright L Corish C The body composition, nutritional knowledge, attitudes, behaviors, and future education needs of senior schoolboy rugby players in Ireland Int J Sport Nutr Exerc Metab 2011 21 5 365 376 10.1123/ijsnem.21.5.365 21799215

